# Mouse SPAG6L, a Key Cytoskeleton Modulator Essential for Male Germ Cell Development, Is Not Required for Sertoli Cell Function

**DOI:** 10.3390/cells14110783

**Published:** 2025-05-26

**Authors:** Tao Li, Wei Li, Cheng Zheng, Jannette M. Dufour, William H. Walker, Shuiqiao Yuan, Zhibing Zhang

**Affiliations:** 1 Institute of Reproductive Health, Tongji Medical College, Huazhong University of Science and Technology, Wuhan 430030, China; 2 Department of Physiology, Wayne State University, Detroit, MI 48201, USAhw1186@wayne.edu (C.Z.); 3 Department of Occupational and Environmental Health, School of Public Health, Wuhan University of Science and Technology, Wuhan 430065, China; 4 Department of Cell Biology and Biochemistry, Texas Tech University Health Sciences Center, Lubbock, TX 79430, USA; 5 Department of Obstetrics, Gynecology and Reproductive Sciences, Magee Womens Research Institute, University of Pittsburgh, Pittsburgh, PA 15260, USA; walkerw@pitt.edu; 6 Laboratory of Animal Center, Huazhong University of Science and Technology, Wuhan 430030, China; 7 Department of Obstetrics & Gynecology, Wayne State University, Detroit, MI 48201, USA

**Keywords:** *Spag6l*, axoneme, central apparatus, cilia, Sertoli cells, spermatogenesis

## Abstract

Mouse sperm-associated antigen 6-like (SPAG6L) evolved from SPAG6, the mammalian ortholog of Chlamydomonas PF16, which is localized in the central apparatus of the motile cilia and is essential for ciliary motility. Even though the amino acid sequences of the two SPAG6 proteins are highly similar, the two proteins have different biological expression patterns in vivo. No major phenotypes were discovered in the global *Spag6* knockout mice. However, the global *Spag6l* knockout mice demonstrated multiple phenotypes in tissues with and without cilia. Since SPAG6L decorates microtubules and modulates cytoskeleton function, and Sertoli cells have a well-developed microtubule transport network, the potential function of SPAG6L in Sertoli cells was evaluated. The floxed *Spag6l* mice were crossed with *Amh-Cre* transgenic mice to inactivate the *Spag6l* gene specifically in Sertoli cells. Surprisingly, the fertility of the homozygous mutant males was not reduced. The testis size and sperm number and motility showed no significant difference to those of the control mice. Testicular histology also showed normal spermatogenesis. No significant changes were observed in the number of Sertoli cells and blood–testis barrier function. Our study showed that the inactivation of only *Spag6l* does not affect Sertoli cell function during the first 6 months of life.

## 1. Introduction

Mammalian sperm-associated antigen 6 (SPAG6) is the ortholog of Chlamydomonas PF16, a protein localized in the central pair of the axoneme that modulates flagellar motility [1]. Mice have two *Spag6* genes. One is the ancient *Spag6*, located on chromosome 2; the other one is an evolved gene called *Spag6-like* (*Spag6l*), located on chromosome 16 [2,3]. The mouse *Spag6* and *Spag6l* genes have different expression patterns. The ancient *Spag6* gene is expressed in the tissues with motile cilia, including brain and testes, while *Spag6l* is widely expressed [4]. Even though the two SPAG6 proteins share a high identity in amino acid sequences, they perform different functions. No major phenotype was discovered in the global *Spag6* knockout mice [4]; however, the global *Spag6l* knockout mice developed significant phenotypes. More than 50% of the global *Spag6l* knockout mice died of hydrocephalus before adulthood, and the surviving males were infertile, not only showing reduced sperm motility but also showing significantly reduced sperm number. The ultrastructure of sperm in the testis and epididymis was disrupted, manifested by frequent loss of sperm heads and disordered flagellum structure, including the loss of a central pair of microtubules and the disorder of the outer dense fibers and fibrous sheaths [5]. Ciliogenesis and ciliary polarity were disrupted in the epithelial cells of the brain ventricles, trachea, middle ear, and eustachian tube in the absence of SPAG6L [5,6].

SPAG6L not only plays a role in the cells with primary cilia and motile cilia, but it also plays a role in the cells without cilia. The immunological synapse is impaired in the immune cells of SPAG6L-deficient mice [7]. Notably, mouse embryonic fibroblasts (MEFs) isolated from *Spag6l*-deficient embryos exhibited diffuse cytoplasmic localization of both F-actin and α-tubulin during migration, contrasting with the polarized distribution pattern observed in wild-type counterparts [8]. These findings collectively suggest that SPAG6L deficiency disrupts normal cytoskeletal architecture and functionality. Moreover, in our previous study [8], we demonstrated complete colocalization of SPAG6L with acetylated tubulin across multiple cell lineages (including MEFs and COS-1 and CHO cells), with their expression levels exhibiting a positive correlation. Tubulin acetylation serves as a critical regulatory mechanism for microtubules, conferring enhanced structural resilience to withstand mechanical stress while also facilitating kinesin-1-mediated cargo transport [9,10]. Our recent in vitro experiments have also revealed partial colocalization between SPAG6L and α-tubulin [3]. Collectively, these findings suggest potential regulatory roles for SPAG6L proteins in cytoskeletal modulation through tubulin-interacting mechanisms.

Spermatogenesis occurs in the testis, which contains germ cells and somatic cells. Even though germ cells are sperm-making cells, somatic cells, including the Sertoli cells and Leydig cells, also regulate the spermatogenesis process [11,12]. Sertoli cells, also known as nurse cells, are the only somatic cells in the seminiferous epithelium. They provide structural support and an immune barrier to germ cells and are involved in various steps of spermatogenesis. They also secrete products to nourish germ cells while engulfing apoptotic germ cells and excess cytoplasm during their development, thereby maintaining the stability of the spermatogenic microenvironment [11,13,14,15]. As dynamic cytoskeletal elements integral to Sertoli cell function, microtubules serve as polarized tracks facilitating the directional transport of developing germ cells during spermatogenesis [15,16,17]. The blood–testis barrier (BTB) is structurally constituted by actin-based tight junctions (TJs) and gap junctions (GJs), in concert with intermediate filament-anchored desmosomes [15,18,19,20]. This tripartite junctional complex plays indispensable roles in both maintaining the homeostatic microenvironment for spermatogenesis and orchestrating the directional migration of germ cells toward the seminiferous tubule lumen.

Given SPAG6L’s putative regulatory effects on both microtubule and F-actin dynamics, we hypothesized its functional involvement in spermatogenesis via the modulation of Sertoli cell cytoskeletal organization. We therefore generated the *Spag6l^flox^*^/flox^ mouse model [21]. To test the model, the floxed mice were crossed with the *Hrpt*-cre mice so that the Spag6l was disrupted globally. The phenotype of the homozygous mutant mice was consistent with that of the global Spag6l knockout mice [21]. The floxed *Spag6l* mice were then crossed with *Amh* (Anti-Müllerian hormone)-Cre mice to specifically inactivate the *Spag6l* in Sertoli cells.

## 2. Materials and Methods

### 2.1. Ethics Statement

The guidelines of the Wayne State University Institutional Animal Care with the Program Advisory Committee (Protocol number: 24-02-6561) were observed in the execution of all animal research.

### 2.2. Sertoli Cell Culture

Sertoli cell line MSC-1 was kindly provided by Dr. Jannette Dufour. It has been shown that the MSC-1 cell line is composed of a single cell type displaying numerous characteristics of Sertoli cells [22]. MSC-1 cells were cultured in Dulbecco modified Eagle medium (DMEM) (Gibco BRL, Gaithersburg, MD, USA) supplemented with penicillin-streptomycin and 5% fetal calf serum (FCS) at 37 °C in a humidified atmosphere of 5% CO_2_/95% air (*v/v*).

### 2.3. Animals and Genotyping

The *Amh-cre* line, originally generated by Dr. Florian Guillou [23], was kindly provided by Dr. William Walker at the University of Pittsburgh. The floxed *Spag6l* line was generated in our laboratory. Genomic DNA was extracted from the toes of 7-day-old mice. Mouse genotypes were identified by PCR using the following primers: *Amh-cre* forward: 5′-GCATTACCGGTCGATGCAACGAGTG-3′; *Amh-cre* reverse: 5′-GAACGTAGAGCCTGT TTTGCACGTTC-3′; *Spag6l* forward: 5′-CTGTGGCATAGTTGTGGGTTTGAAG-3′; *Spag6l* reverse: 5′-CAGCAGAGCCTGTAGCTCTACTC-3′.

### 2.4. RT-PCR

Total RNA was extracted from Sertoli cells and mouse testes using TRIzol (Invitrogen, Waltham, MA, USA). cDNA was synthesized by reverse transcription using a cDNA synthesis kit (Meridian Bioscience, Cincinnati, OH, USA). RT-PCR was performed with cDNA as a template using the following specific primers: *Spag6* forward: 5′-CCATCACAAACACGTTGCCCGT-3′; *Spag6* reverse: 5′-GTTAATAAGAGGCTGATAGCTG-3; *Spag6l* forward: 5′-GTGGCTGTCACAAACACGCTGC-3′; *Spag6l* reverse: 5′-CAGTGGTTGGTAGCTGTCCACC-3′.

### 2.5. Histological Examination

Mouse testicular and epididymal tissues were collected and then fixed overnight using Bouin’s solution (SIGMA, Saint Louis, MO, USA, HT10132). The tissues were then embedded in paraffin, and 5-micron paraffin sections were prepared. After removing the paraffin, standard hematoxylin–eosin staining (H&E) and Periodic Acid–Schiff (PAS) stains were used for histological analysis. The sections were mounted with neutral gum and then photographed.

### 2.6. Male Fertility Test

In this study, 2-month-old and 5-month-old cKO male mice and control mice were bred independently with 2-5-month-old WT female mice for one month, and vaginal plugs were recorded to verify the occurrence of mating. The number and survival of offspring were also recorded, and the average number of litters was used to assess fertility.

### 2.7. Immunofluorescence Staining

After the testes were collected, they were immediately fixed in 4% Formalin overnight and then embedded in paraffin. Furthermore, 5-micron sections were prepared using a Leica paraffin slicer, then dewaxed and boiled in 10 mM Tris-EDTA (pH  =  9.0) or 10 mM sodium citrate (pH 6.0) for 30 min for antigen retrieval. Then, the sections were blocked with 10% goat serum at room temperature for one hour, followed by overnight incubation with the corresponding primary antibodies. The next day, PBS was used to wash away the excess primary antibodies, followed by incubation with the secondary antibodies at room temperature for one hour. For PNA staining, following blocking with 10% (*v/v*) goat serum, samples were incubated with Alexa Fluor 488-conjugated PNA (1:4000 dilution in 10% goat serum) for 1 h at room temperature under light-protected conditions. After washing away the excess secondary antibodies (or PNA) with PBS, a mounting medium containing DAPI nuclear dye was added, and images were taken using a fluorescence microscope. The antibodies or dye used in this study are as follows: WT1 (Proteintech, 12609-1-AP, Dilution ratio: 1:200); DDX4 (Abcam, Cambridge, UK, ab27591, Dilution ratio: 1:400); ZO-1 (Thermofisher, Carlsbad, CA, USA 33-9100, Dilution ratio: 1:200); Connexin 43 (Cell Signaling Technology, Danvers, MA, USA, 3512S, Dilution ratio: 1:200); PNA-488 (Thermo Scientific, Carlsbad, CA, USA, P5767580, Dilution ratio: 1:4000); Goat Anti-Mouse IgG H and L (Alexa Fluor^®^ 488) (Abcam, Cambridge, UK, ab150113, Dilution ratio: 1:2000); Goat Anti-Rabbit IgG H and L (Alexa Fluor^®^ 555) (Abcam, Cambridge, UK, ab150078, Dilution ratio: 1:2000); DAPI (Vector Labs, Burlingame, CA, USA); Phalloidin (Thermo Scientific, Carlsbad, CA, USA, A12379).

### 2.8. Cell Counts

ImageJ 1.54d was used to analyze the images to count the number of Sertoli cells and germ cells. To count the cell numbers of the seminiferous tubule, at least 20 tubules were analyzed for each testis per mouse.

### 2.9. Sperm Counting

The cauda epididymis was promptly removed and immersed in PBS solution maintained at 37 °C. The tissue was then incised, allowing the spermatozoa to disperse. Following this, a tenfold dilution of the sample was prepared, and the diluted sample was carefully dispensed onto a cell counting plate for enumeration. Sperm concentration was calculated using the formula: (number of sperm counted/volume of diluted sample) × dilution factor.

### 2.10. Isolation of Sertoli Cells from Testis and Labeling of F-Actin with Phalloidin

Testes were aseptically dissected from the mice, and the tunica albuginea was removed. Seminiferous tubules were enzymatically digested in DMEM/F12 medium supplemented with 2 mg/mL collagenase, 20 μg/mL DNase I, and 2 mg/mL hyaluronidase (all from Sigma) at 37 °C for 30 min to generate a single-cell suspension. The suspension was filtered through a 70-μm cell strainer (Falcon, Corning, NY, USA) to remove undigested tissue fragments. Cell density was quantified using a hemocytometer, and cells were seeded at 50,000 cells/cm^2^ in DMEM/F12 medium (Gibco) containing 10% fetal bovine serum (FBS; HyClone, Logan, UT, USA) and 1% penicillin-streptomycin (Thermo Fisher, Waltham, MA, USA). Cultures were maintained in a humidified incubator at 35 °C with 5% CO₂ for 72 h. On day 4, adherent Sertoli cell monolayers were washed twice with PBS, followed by hypotonic treatment with 20 mM Tris-HCl (pH 7.5; MilliporeSigma, Darmstadt, Germany) at room temperature for 5 min to selectively remove residual germ cells. Isotonicity was immediately restored using fresh medium replacement, and purified Sertoli cells were further cultured for downstream applications. Sertoli cells were plated on 8-well chamber slides (Thermo Scientific Nunc™) pre-coated for immunofluorescence assays. After removing the culture medium, cells were washed twice with PBS. Fixation was performed with 4% paraformaldehyde (PFA; Sigma-Aldrich) at room temperature (RT) for 15 min, followed by two additional PBS washes. Cellular permeabilization was achieved using 0.1% Triton X-100 (Sigma-Aldrich) in PBS for 15 min at RT. Post-permeabilization washes (×2 PBS) preceded incubation with Alexa Fluor™ 488-conjugated phalloidin (1:40 dilution in 1% BSA/PBS; Invitrogen, Carlsbad, CA, USA) for 1 h at RT under light-protected conditions. Following final PBS washes (×2), nuclei were counterstained with DAPI (Vector Labs) and imaged on a Nikon DS-Fi2 Eclipse 90i Motorized Upright Fluorescence Microscope (The C.S. Mott Center for Human Growth and Development, Department of Obstetrics and Gynecology, Wayne State University).

### 2.11. Protein Extraction and Western Blot Analysis

Testicular tissues were aseptically dissected from C57BL/6 mice and homogenized in RIPA lysis buffer supplemented with protease inhibitor cocktail (Roche, Basel, Switzerland). Protein concentrations were quantified using a bicinchoninic acid (BCA) assay kit (Bio-Rad Laboratories, Hercules, CA, USA) according to the manufacturer’s protocol. Equal amounts of protein lysates (30 μg per lane) were resolved by 10% SDS-PAGE under reducing conditions and subsequently transferred onto polyvinylidene difluoride (PVDF) membranes using a wet transfer system at 4 °C. Membranes were blocked for 1 h at room temperature with 5% non-fat milk dissolved in Tris-buffered saline containing 0.1% Tween-20 (TBST). Primary antibodies were shaken overnight at 4 °C with gentle agitation using the following dilutions in blocking buffer: anti-GATA1 (Santa Cruz, sc-265, 1:500) and anti-GAPDH (Santa, Dallas, TX, USA, sc-47724, 1:1000). After three 10-min TBST washes, membranes were incubated with species-matched horseradish peroxidase (HRP)-conjugated secondary antibodies (Proteintech, Rosemont, IL, USA, SA00001-1 and SA00001-2) diluted 1:2000 in blocking buffer for 1 h at room temperature. Following extensive TBST washing, immunoreactive bands were visualized using an enhanced chemiluminescence (ECL) substrate (Lamda Biotech; components A and B mixed at a 1:1 ratio) and captured using a Bio-Rad ChemiDoc MP imaging system with automatic exposure optimization.

### 2.12. Statistical Analysis

Statistical analyses were performed using Student’s *t*-test. *p < 0.05 was considered significant. Graphs were created using Microsoft Excel and GraphPad Prism v9.0.0.121.

## 3. Results

### 3.1. Spag6l mRNA Is Expressed in Sertoli Cells

The MSC-1 cell line is a Sertoli cell line derived from transgenic mice carrying a fusion gene construct comprising the human Anti-Müllerian hormone (AMH) transcriptional regulatory sequence fused to the SV40 T-antigen gene [24]. This cell line has been used by many researchers as an in vitro model of Sertoli cells [25,26]. Total RNA was isolated from MSC-1 cells using TRIzol reagent, followed by reverse transcription into cDNA. RT-PCR analysis revealed high-level expression of both *Spag6* and *Spag6l* in the MSC-1 cell line (Supplementary Figure S1A), with testicular cDNA serving as a positive control.

### 3.2. Generation of Sertoli Cell-Specific Spag6l Knockout Mice

Using CRISPR/Cas9 technology, we inserted LoxP sites flanking exon 3 of the *Spag6l* gene [21]. The subsequent breeding of LoxP-modified mice with *Amh*-Cre transgenic mice enabled the Cre recombinase-mediated excision of exon 3 between the paired LoxP sites, generating a conditional knockout allele specifically in Sertoli cells (Supplementary Figure S1B). Genotyping analysis confirmed the *Spag6l^flox/flox^* homozygous and *Spag6l^flox/+^* heterozygous genotypes, as shown in Supplementary Figure S1C. The *Spag6l^flox/flox^; Amh-Cre*^+^ mice were considered to be homozygous conditional knockout (cKO) mice. The *Spag6l^flox/+^* mice, the *Amh-Cre*^+^ or *Spag6l^flox/flox^* mice, and the *Amh-Cre*^−^ mice were analyzed as the controls.

### 3.3. The cKO Male Mice Had Normal Fertility with Normal Testis Size and Sperm Number and Motility

**Figure 1:**
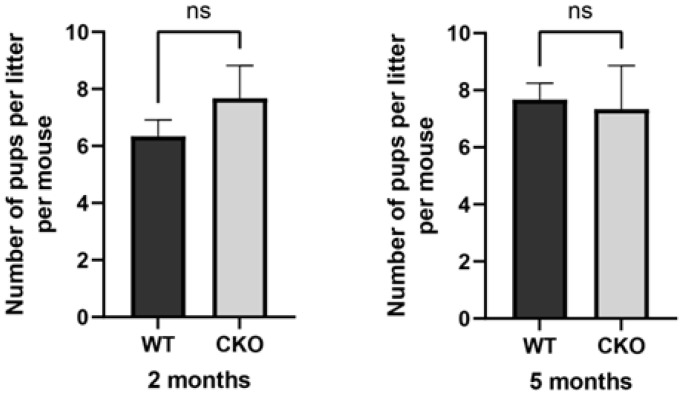
*Spag6l* cKO mice were fertile. Longitudinal assessment of male reproductive capacity. Age-stratified cohorts (2- and 5-month-old) of conditional knockout (cKO) and age-matched WT male mice underwent continuous mating trials with proven-fertility WT females. Litter size quantification revealed maintained reproductive performance in cKO mice across both age groups (2-month: WT 6.3 ± 0.6 vs. cKO 7.7 ± 1.6; 5-month: WT 7.7 ± 0.6 vs. cKO 7.3 ± 1.5, mean ± SD). Statistical analysis by two-tailed unpaired Student’s *t*-test (*n* = 3 biological replicates per group, ns: *p* > 0.05).

cKO mice exhibited no overt phenotypic differences compared to WT controls, including body weight and developmental progression. To investigate whether Sertoli cell-specific deletion of *Spag6l* impacts male fertility, 2- and 5-month-old cKO males underwent monthly fertility assessments over a 3-month breeding trial. Surprisingly, all experimental cohorts demonstrated reproductive competence equivalent to that of the controls (Figure 1), with no statistically significant differences in mean litter size per mating pair (*p* > 0.05).

**Figure 2:**
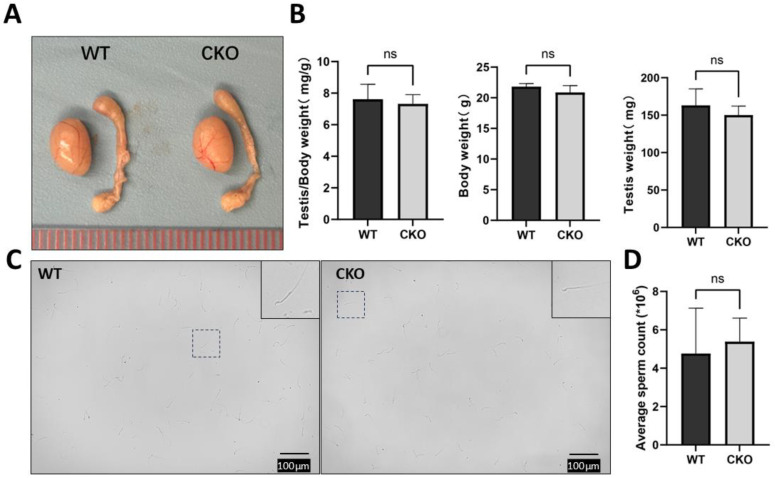
Comprehensive evaluation of testis morphology and spermatogenic capacity. (**A**) Gross morphological analysis. Comparative visualization of testes and epididymes from 2-month-old cKO mice versus WT littermates. (**B**) Testis weight index quantification. Combined testicular mass (bilateral, mg/g) normalized to body weight showed comparable ratios between genotypes (WT: 7.32 ± 0.59 vs. cKO: 7.62 ± 0.95, mean ± SD). (**C**) Spermatozoa morphological assessment. Caudal epididymal sperm suspensions obtained through mechanical dissociation in PBS (37 °C, 20 min) exhibited normal sperm architecture in both groups (inset: representative contrast micrograph). (**D**) Sperm counting. Hemocytometric analysis revealed conserved sperm concentrations in cKO mice (WT: 4.77 ± 2.36 ×10⁶/mL vs. cKO: 5.39 ± 1.22 ×10⁶/mL). Statistical significance determined by two-tailed unpaired Student’s *t*-test (*n* = 3 biological replicates, ns: *p* > 0.05).

As Sertoli cells are pivotal regulators of testicular morphogenesis, we systematically evaluated whether Sertoli cell-specific deletion of *Spag6l* compromises testicular functionality (Figure 2). Comparative analyses of age-matched cKO and WT littermates revealed no significant differences in testis/body weight ratio (Figure 2A,B), indicating preserved testicular development despite *Spag6l* deletion. Subsequently, cauda epididymal spermatozoa were collected from sexually mature cKO males (*n* = 5/group, age: WT: 63 days ± 11.70 vs. cKO 67.4 days ± 14.17) for quantitative assessment. Quantification using a hemocytometer demonstrated comparable sperm counts between genotypes (Figure 2C,D). In addition, there were no morphological changes observed in the sperm released from the mouse epididymis (Supplementary Figure S2). These findings collectively suggest that Sertoli cell-specific *Spag6l* deficiency does not overtly impair spermatogenic output.

### 3.4. Spermatogenesis Was Normal in the Spag6l cKO Mice

**Figure 3:**
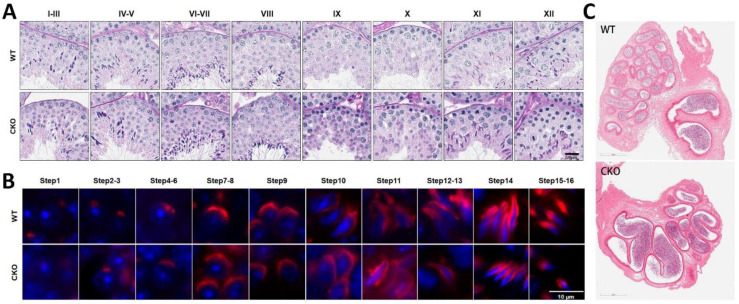
Histomorphometric evaluation of germ cell development and spermatogenic progression. (**A**) Stage-specific seminiferous epithelium analysis. Periodic Acid–Schiff (PAS) histochemistry revealed preserved cytoarchitectural organization across all spermatogenic stages (I–XII) in 2-month-old cKO mice, with intact BTB formation and normal germ cell associations. Scale bar: 20 μm. (**B**) Spermiogenesis phase tracking: Immunofluorescence demonstrated characteristic 16-step spermatogenic progression, visualized through lectin-based acrosomal tracking (PNA, red) and nuclear demarcation (DAPI, blue). Scale bar: 10 μm (**C**) H&E staining of the caudal epididymis of 2-month-old mice. No significant change in sperm number was found. Scale bar in the corner: 300 μm.

The principal function of the reproductive system resides in the generation of germ cells. Sertoli cells play an essential role in the regulation of spermatogenesis [15]. Henceforth, we scrutinized the process of spermatogenesis within the testicular tissue (Figure 3). Briefly, histological sections of adult testes and epididymides were meticulously prepared and examined. In the testicular tissue of cKO mice, distinct phases of the 12-stage seminiferous epithelial cycle and the 16-step spermiogenesis process were unequivocally discernible (Figure 3A,B and Supplementary Figure S3). Furthermore, H&E staining of the epididymal sections revealed a substantial aggregation of mature spermatozoa within the lumen (Figure 3C). These results indicate that deletion of *Spag6l* in Sertoli cells may not affect the normal spermatogenesis process.

### 3.5. The Disruption of Spag6l in Sertoli Cells Did Not Change the Population of Germ Cells and Sertoli Cells in the Seminiferous Tubules

**Figure 4:**
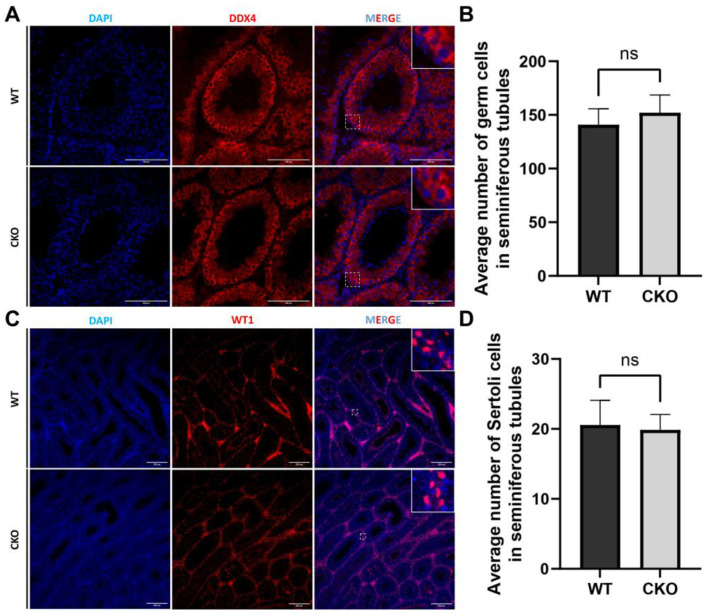
Immunofluorescence showed no obvious changes in germ cells and Sertoli cells. (**A**) Paraffin section of testis showing the location of germ cells in the seminiferous tubules. Scale bar: 100 μm. (**B**) Quantification of germ cells in seminiferous tubules. *n* = 20; WT: 140.90 ± 14.86 vs. cKO: 152.20 ± 16.60. (**C**) Location of Sertoli cells in seminiferous tubules. Scale bar: 100 μm. (**D**) Average number of Sertoli cells in seminiferous tubules. *n* = 20; WT: 20.55 ± 3.53 vs. cKO: 19.85 ± 2.20. DDX4: germ cell marker; WT1: Sertoli cell marker; DAPI: nuclear marker. All data are from tissue from 2-month-old mouse testes. Statistical significance determined by two-tailed unpaired Student’s *t*-test (*n* = 3 biological replicates, ns: *p* > 0.05).

To investigate potential alterations in testicular cellular composition induced by *Spag6l* deficiency, we conducted immunofluorescence characterization of germ cell and Sertoli cell populations within seminiferous tubules (Figure 4). Germ cells were identified using the meiotic marker DDX4 (cytoplasmic localization in spermatogonia, spermatocytes, and round spermatids), while Sertoli cells were distinguished by WT1 nuclear staining. Quantitative analysis of cross-sectional tubule profiles revealed comparable germ cell populations between genotypes, with cKO mice showing no statistically significant changes in the mean number of DDX4^+^ cells per tubular lumen cross-section (Figure 4A). Similarly, WT1^+^ Sertoli cells maintained their characteristic basal compartment localization in cKO testes, with cell counts remaining comparable to those of WT controls (Figure 4B).

### 3.6. The Sertoli Cells Maintain a Normal Cellular Junction in the Absence of SPAG6L

**Figure 5:**
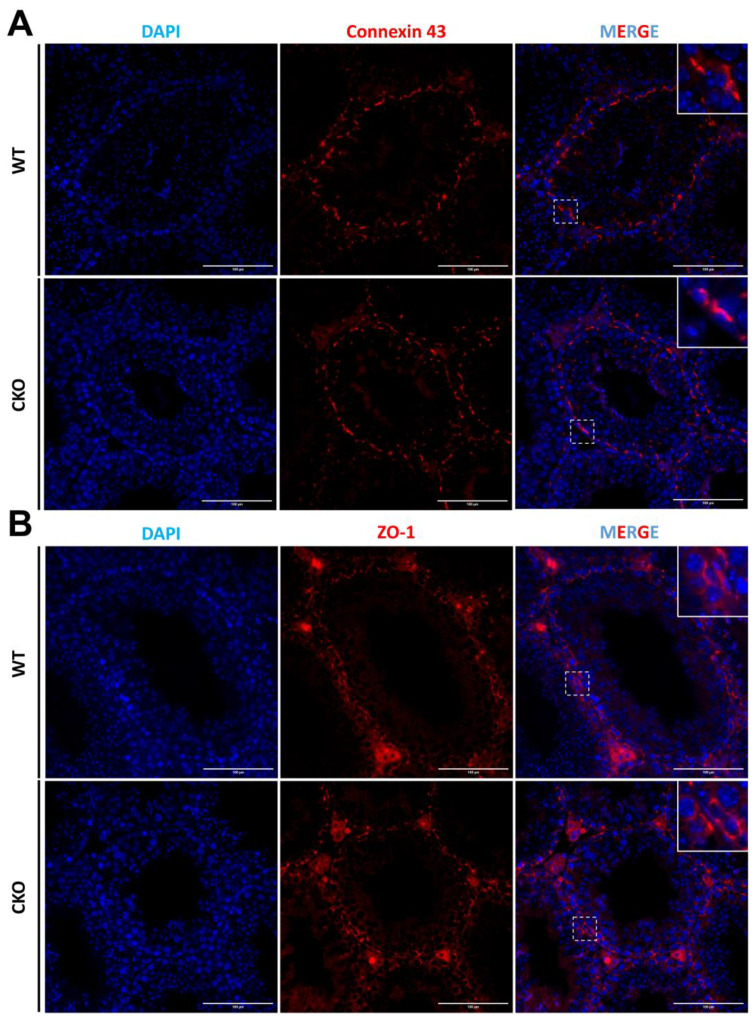
Immunofluorescence showed that the structure of testicular BTB was unchanged. (**A**) ZO-1 was highly enriched in the lateral membrane area (close to the basement membrane) of Sertoli cells in the seminiferous epithelium of cKO mice, and the signal was continuous without abnormal localization, indicating that there was a complete tight junction structure between Sertoli cells. Scale bar: 100 μm. (**B**) In the seminiferous epithelium of cKO mice, Connexin 43 signals were mainly localized on the basal side, indicating gap junctions formed between Sertoli cells. Connexin 43 signals were also observed in the central area close to the lumen, suggesting the formation of gap junctions between supporting cells and germ cells. The above results were not significantly different from the WT. Scale bar: 100 μm. ZO-1: Tight junction-related protein. Connexin 43: Gap junction-related protein. DAPI: nuclear marker.

Within the seminiferous tubules, Sertoli cells play a pivotal role in establishing the essential blood–testis barrier (BTB). Hence, we employed antibodies against ZO-1 and Connexin 43 to delineate two crucial intercellular junction types within the BTB, namely tight junctions (TJs) and gap junctions (GJs). Our observations revealed that the distribution and integrity of these junctions remained unaffected when *Spag6l* was disrupted in the Sertoli cells (Figure 5).

### 3.7. The Cytoskeleton of Sertoli Cells Shows Normal Structure in the Absence of SPAG6L

**Figure 6:**
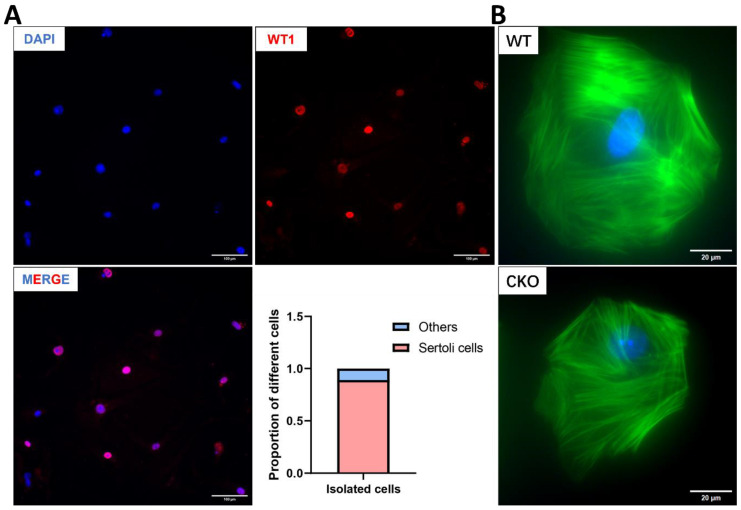
Cytoskeletal integrity assessment in isolated Sertoli cells. (**A**) Validation of cellular purity. WT1 immunostaining (red: Sertoli cell nuclear marker) with DAPI counterstain (blue: pan-nuclear) confirms efficient Sertoli cell isolation. Scale bar: 100 μm. (**B**) Microfilament network analysis. Phalloidin staining (green: F-actin) reveals preserved cytoskeletal architecture in cKO Sertoli cells. DAPI (blue) denotes nuclei. Tissues derived from postnatal day 20 mice. Scale bars: 20 μm.

As a crucial structural element of the Sertoli cell cytoskeleton, F-actin plays a pivotal role in preserving BTB integrity. The actin-based microfilament network not only encapsulates developing germ cells to facilitate their directional migration through the seminiferous tubule but also coordinates with gap junctions, adherens junctions, and other intercellular connectors to mediate Sertoli–germ cell communication [27,28]. To investigate potential *Spag6l* deficiency-induced alterations in Sertoli cell F-actin organization, we performed primary Sertoli cell isolation from WT and cKO mouse testes (Figure 6). Cellular purity was confirmed through WT1 nuclear immunostaining, while phalloidin staining revealed F-actin cytoskeletal architecture. Notably, comparative analysis demonstrated no significant differences in F-actin organization between WT and cKO Sertoli cells (Figure 6B).

### 3.8. Discussion

Mouse SPAG6L is a protein that evolved from SPAG6. SPAG6L not only conducts the conserved function as a central apparatus protein to control ciliary motility but also plays an essential role in cilia formation [5]. In global *Spag6l* knockout mice that survived to adulthood, infertility emerges as the cardinal phenotypic manifestation, attributable to asthenozoospermia with structural flagellar abnormalities [4]. During evolution, SPAG6L gained new functions, and we believe that the key mechanism involves the modulation of microtubule/cytoskeleton functions by regulating tubulin acetylation levels [8,29]. Our in vitro experiments [3,8] revealed SPAG6L’s potential regulatory roles in modulating both F-actin and α-tubulin dynamics. Concurrently, given the critical involvement of Sertoli cell cytoskeletal regulation in maintaining the BTB and facilitating directional germ cell transport [11,15], we were interested in investigating the functional significance of SPAG6L within Sertoli cells.

However, systematic phenotyping reveals that *Spag6l* ablation does not exert significant detrimental effects on Sertoli cell functional homeostasis during the first 6 postnatal months. cKO mice exhibited normal fertility, with no significant differences in litter size or sperm concentration. Histological evaluation via Periodic Acid–Schiff (PAS) staining further confirmed intact seminiferous epithelial architecture at all stages of the spermatogenic cycle of cKO mice. Immunofluorescence revealed conserved germ cell and Sertoli cell populations within the seminiferous tubules of cKO mice, also demonstrating the location of gap junction protein Connexin 43 and tight junction-associated protein ZO-1, indicating intact Sertoli cell junctional functionality. Furthermore, phalloidin-based F-actin visualization in isolated Sertoli cells showed conserved cytoskeletal architecture, with filament orientation indistinguishable from controls. We also examined testicular GATA-1, another Sertoli cell marker [11] by Western blot analysis. The level was also normal in the cKO mice (Supplementary Figure S4). The lack of observable phenotypes in our study could arise from two compensatory mechanisms: (1) the inherent absence of ciliary architecture in terminally differentiated Sertoli cells, which minimizes dependency on SPAG6L, and (2) functional redundancy provided by persistent SPAG6 expression (93% sequence homology with SPAG6L in mouse models). Our initial experimental strategy sought to delineate the differential expression profiles of SPAG6 and SPAG6L through immunofluorescence microscopy or immunoblotting assays. However, this endeavor encountered formidable technical challenges due to the unavailability of high-specificity commercial antibodies capable of distinguishing them [30,31]. Despite the generation of six distinct polyclonal antibodies targeting divergent epitopes, there is insufficient specificity for unambiguous discrimination. These technical limitations currently preclude definitive subcellular localization analysis of SPAG6L in Sertoli cells.

Given that *Spag6*^−/−^ has been reported to generate no obvious abnormalities in multiple systems, including the reproductive system [3], it may be important to further study the functions of these two *Spag6* genes in Sertoli cells. A potential solution is to cross existing *Spag6*^−/−^ mice with our *Spag6l*^flox/flox^; Amh-Cre^+^ mouse line to generate triple-transgenic *Spag6*^−/−^; *Spag6l*^flox/flox^; Amh-Cre^+^ animals. This strategy would complete the double knockout of *Spag6* and *Spag6l* in Sertoli cells and allow exploration of the synergistic/antagonistic effects of *Spag6* and *Spag6l* in Sertoli cells. Alternatively, we recently initiated work to establish a knock-in mouse model, adding HA and V5 tag proteins after SPAG6 and SPAG6L, respectively, which is expected to further reveal the similarities and differences in their expression and localization in Sertoli cells in future studies.

Collectively, our findings demonstrate that the conditional deletion of *Spag6l* specifically in Sertoli cells does not compromise their intrinsic physiological functions nor perturb normal spermatogenesis. This phenotype may be attributed to the functional redundancy conferred by the preserved expression of its paralog *Spag6*. These data provide critical insights into the cell type-specific functional hierarchy of *Spag6* family members within the testicular microenvironment. Building upon previous studies in globe knockout models [3,4], our results reinforce the notion that *Spag6l* exerts more prominent roles in regulating germ cell functions rather than somatic Sertoli cell functions.

## Data Availability

All relevant data are included in the article and its Supplementary Materials.

## Supplementary Materials

The following supporting information can be downloaded at https://www.mdpi.com/article/10.3390/cells14110783/s1, Figure S1: Validation of Spag6l expression and generation of Spag6l conditional knockout mice. Figure S2: Epididymic sperm morphology examination. Figure S3: PAS stain of 5-month testis. Figure S4: Western blot results of GATA-1.
