# Supplementary material for: Mouse SPAG6L, a Key Cytoskeleton Modulator Essential for Male Germ Cell Development, Is Not Required for Sertoli Cell Function
Source: Cells. 2025 May 26;14(11):783. doi: 10.3390/cells14110783 (PMC12153848; doi:10.3390/cells14110783)
Supplement: Supplementary file 1 [file cells-14-00783-s001.zip › cells-3616212-supplementary.pdf]

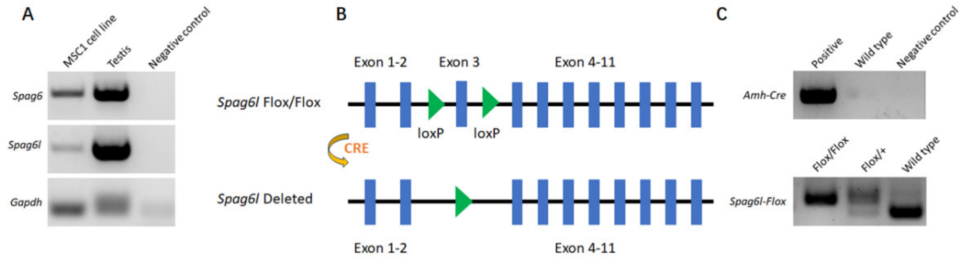

Figure S1: Validation of *Spag6l* expression and generation of *Spag6l* conditional knockout mice. (A) RT-PCR profiling of *Spag6*/*Spag6l* transcripts in MSC-1 Sertoli cells. Both genes exhibited expression with *Gapdh* serving as the loading control. (B) Targeted deletion strategy for *Spag6l* conditional knockout. The loxP-flanked exon 3 undergoes Cre-mediated recombination, generating a frameshift mutation. (C) Genotypic validation. Representative electrophoretic profiles confirm successful targeting in Flox/Flox (Fl/Fl) and knockout (cKO) models;

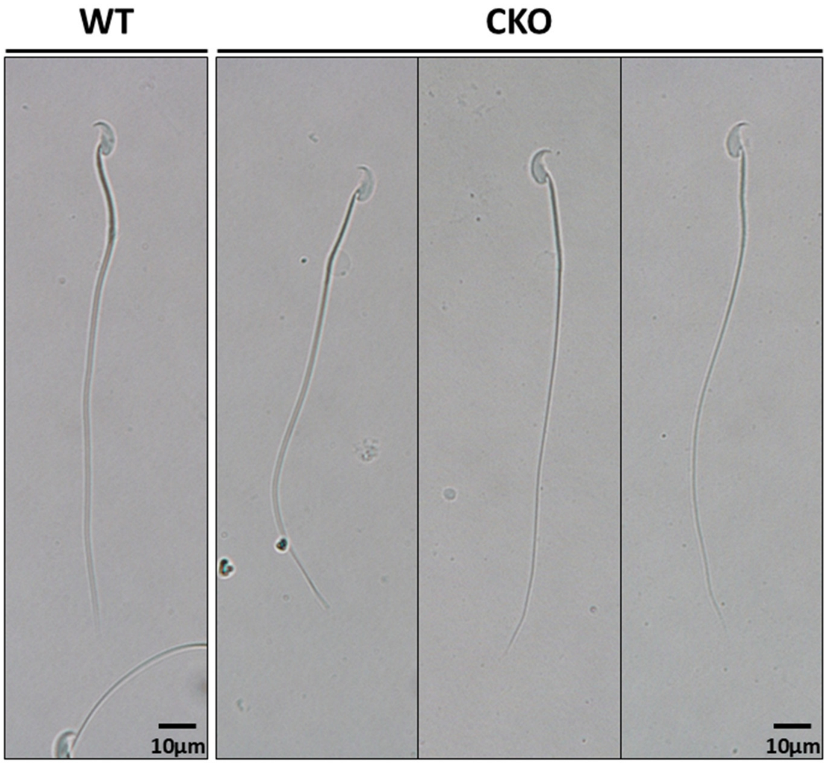

Figure S2: Epididymic sperm morphology examination. The morphology of epididymal sperm in cKO mice and wild-type control mice was observed under a 40x microscope, and the results showed that there were no significant abnormalities in cKO mice;

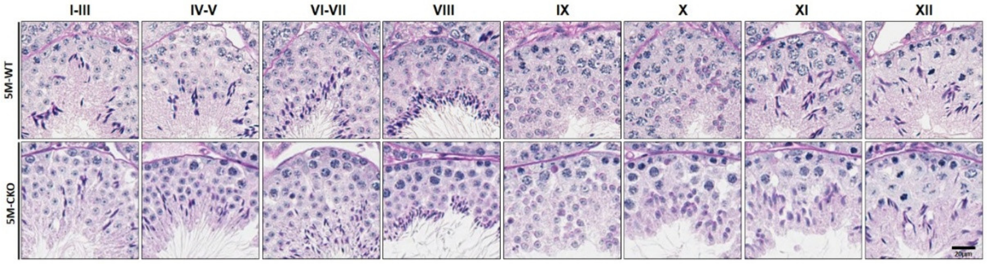

Figure S3: PAS stain of 5-month testis. There are no significant differences between WT and cKO

mice at 12 stages of spermatogenesis;

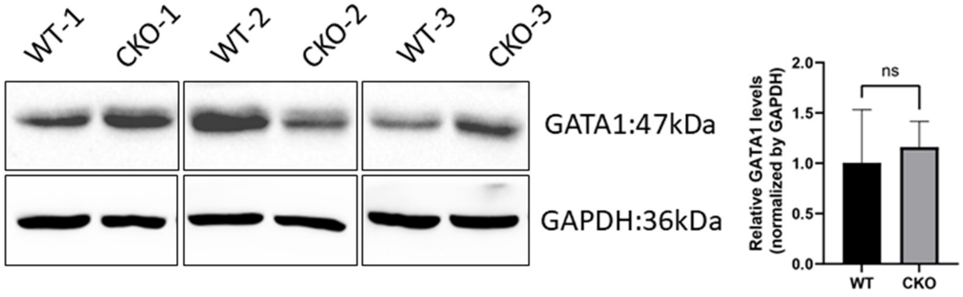

Figure S4: Western blot results of GATA-1. Testicular samples from 3 pairs of 2-month-old biological replicates were selected for western blot analysis. ImageJ software was used to calculate grayscale values, and GATA-1 expression levels were normalized to GAPDH. The results demonstrated no significant change in GATA1 protein expression levels in cKO mouse testes.
